# BlendNet: a blending-based convolutional neural network for effective deep learning of electrocardiogram signals

**DOI:** 10.3389/frai.2025.1625637

**Published:** 2025-08-22

**Authors:** S. Premanand, Sathiya Narayanan

**Affiliations:** School of Electronics Engineering (SENSE), Vellore Institute of Technology, Chennai, India

**Keywords:** electrocardiogram, convolution neural network, scalogram, image blending, binary image

## Abstract

**Introduction:**

In recent years, Deep Learning (DL) architectures such as Convolutional Neural Network (CNN) and its variants have been shown to be effective in the diagnosis of cardiovascular disease from ElectroCardioGram (ECG) signals. In the case of ECG as a one-dimensional signal, 1-D CNNs are deployed, whereas in the case of a 2D-represented ECG signal, i.e., two-dimensional signal, 2-D CNNs or other relevant architectures are deployed. Since 2D-represented ECG signals facilitate better feature extraction, it is a common practice to convert an ECG signal into a scalogram image using a continuous wavelet transform (CWT) approach and then subject it to a DL architecture such as 2-D CNN. However, this traditional approach captures only a limited set of features of ECG and thereby limits the effectiveness of DL architectures in disease detection.

**Methods:**

This work proposes “BlendNet,” a DL architecture that effectively extracts the features of an ECG signal using a blending approach termed “alpha blending.” First, the 1-D ECG signal is converted into a scalogram image using CWT, and a binary version of the scalogram image is also obtained. Then, both the scalogram and binary images are subjected to a sequence of convolution and pooling layers, and the resulting feature images are blended. This blended feature image is subjected to a dense layer that classifies the image. The blending is flexible, and it is controlled by a parameter α, hence the process is termed as alpha blending. The utilization of alpha blending facilitates the generation of a composite feature set that incorporates different characteristics from both the scalogram and binary versions.

**Results:**

For experiments, a total of 162 ECG recordings from the PhysioNet database were used. Experimental results and analysis show that, in the case of α = 0.7, BlendNet's performance surpasses the performance of (i) traditional approaches (that do not involve blending) and (ii) state-of-the-art approaches for ECG classification.

**Discussion:**

Experimental outcomes show that the proposed BlendNet is flexible regarding dense layer settings and can accommodate faster alternatives [i.e., machine learning (ML) algorithms] for faster convergence. The superior performance at α = 0.7 indicates that alpha blending allows for richer composite feature sets, leading to improved classification accuracy over conventional feature extraction and classification methods.

## 1 Introduction

Deep Learning-based diagnosis of cardiovascular disease from ECG signals involves two major steps: pre-processing (Safdar et al., 2024) and classification (Wu and Guo, 2025). The pre-processing step generally involves resizing and filtering of signals. The classification step involves feature extraction and signal categorization. Popular DL architectures like CNN and its variants have shown significant results in the medical domain, especially with ECG. In the case of ECG as a one-dimensional signal, 1D-CNNs are deployed. These models are enhanced and contributed in many ways: like combining with Leaky-ReLU (Lakhdari and Saeed, 2022) activation function, an enhanced model for extracting signals from paper-based ECG data (Nguyen et al., 2022), with real and noise-attenuated ECG signals (Ahmed et al., 2023), for robust classification 1-D Convolutional deep residual neural networks (Khan et al., 2023) utilized, and even explored in authentication (Yuniarti et al., 2024). Apart from different scenarios, ECG signals can be combined with different advanced CNN variants such as Deep-CNN (Li et al., 2021), SE-ResNet152 (Xu et al., 2021), MobileNetV2 (Cordos et al., 2021), ResNet152V2, DenseNet169, COV-ECGNET (Rahman et al., 2022), MobileNetV2 combination with BiLSTM (Shin et al., 2022), and InceptionV3 (Bhosale and Patnaik, 2023) and showed promising results. In these architectures, ECG signals are subjected to a sequence of convolution and pooling layers for feature extraction and then to a dense neural network layer for decision-making (i.e., classification). In most of the ECG classifications, scalogram images are utilized from the signal by CWT approach, for extracting non-linear and non-stationary features (Gupta et al., 2021), image classification by representing image over the time-frequency domain (Kim, 2021), extracting R peak and RR interval features (Wang et al., 2021), statistical parameters (Alharbey et al., 2022), classification with various CNN variants for better results (Dessai and Virani, 2023), with RGB image classification from scalogram (Kumar and Ramachandran, 2023), for collecting multi-spectral information (Mewada, 2023), and for classification with transformers (Qiu et al., 2024). In one of the cases, the binarized version of the scalogram image is used for classification (Naz et al., 2021).

Owing to the fact that each version of the image contains some unique features, in this work, we propose an approach termed “alpha blending' which blends the features extracted from the scalogram and its binarized version through a sequence of convolution and pooling layers. The blended feature map is subjected to a dense neural network layer (as in traditional CNN) that classifies the image. This proposed architecture is termed as BlendNet. The blending step is flexible as it involves a parameter α. For experiments, a total of 162 ECG recordings from the PhysioNet database were used. There were three categories of patients: Congestive Heart Failure (CHF), Cardiac Arrhythmias (ARR), and Normal Sinus Rhythms (NSR). There are 36 recordings from people with NSR, 30 with people with CHF, and 96 with ARR. The objective of the proposed approach is to classify ARR, CHF, and NSR. The experimental results shows that the BlendNet architecture achieves the best performance α = 0.7, and it outperforms non-blending approaches. The proposed BlendNet is flexible in terms of dense layer settings, as it can accommodate any complicated dense layers, for example, the dense layers in InceptionV3, ResNet152V2, DenseNet169, or MobileNetV2. In applications where execution speed is of utmost concern, the dense layer can be replaced with faster alternatives (i.e., ML algorithms) for faster convergence.

Notable works in literature relevant to this problem statement are the DL framework in Prusty et al. (2024) which utilizes Scale Invariant Feature Transform (SIFT) based features for detecting heart failures and the framework in Saeed and Yousif (2021) which utilizes the slantlet based statistical features. Both these approaches extract features from the PhysioNet ECG data and apply a DL architecture. Although they have exhibited good classification performance, they depend on a single paradigm for feature extraction. On the other hand, the BlendNet framework proposed in this manuscript deploys a blending framework to form a composite feature map, which is then subjected to a classification architecture/algorithm.

The contributions of this manuscript are as follows:

- A novel DL architecture termed as BlendNet which involves flexible blending of image features using alpha blending.
- An ablation study to emphasize the importance of alpha blending in the proposed BlendNet.
- A flexibility study to explore the choices of dense layer settings for the classification task in the proposed BlendNet.
- A faster alternative for BlendNet, which incorporates the computationally efficient ML algorithms in place of the dense layer.

The remaining sections are structured as follows. Section 2 presents a survey of several ML and DL architectures associated with diverse ECG data. Section 3 provides a detailed explanation of the proposed BlendNet and presents an analysis of its computational complexity. Section 4 presents the experimental validation of the proposed architecture and establishes a comparison with the state-of-the-art. Section 5 concludes the paper with recommendations for future research.

## 2 Literature survey

Recent research has shown substantial progress in identifying irregular heart rhythms using CNNs. Ahmed et al. (2023) employed a 1-D CNN to classify four distinct categories in the MIT-BIH dataset and achieved a remarkable accuracy of 99%. In another work (Lakhdari and Saeed, 2022), including the LeakyReLU activation function in 1-D CNN architectures on the same dataset results in accuracies ranging from 97% to 99%. A modified version of the 1-D CNN called SEResNet18 was used in a dataset of ECG images containing data from cardiac and COVID-19 patients. The model achieved accuracies of 98.42% to distinguish COVID-19 cases from normal cases and 98.50% to distinguish COVID-19 cases from other classes. In particular, the model successfully extracted signals from the scanned ECG records (Khan et al., 2021; Nguyen et al., 2022). There is another progress where a 2-D CNN achieved an accuracy of 99.52% on the MIT-BIH dataset by combining wavelet-based spectral features with CNN's temporal features. This demonstrates the effective combination of advanced signal processing techniques and DL. These advances highlight the strong flexibility and growing accuracy of CNN models in diagnosing heart conditions using different datasets and architectural improvements.

Many studies associated with ECG have recently been conducted in 2-D format, mainly because of extracting morphological features (Wang et al., 2021), non-linear and non-stationary features (Gupta et al., 2021), comparing images with signals (Kim, 2021), for statistical features (Alharbey et al., 2022), good performance across different CNN variants (Dessai and Virani, 2023), performing well in other imaging like RGB (Kumar and Ramachandran, 2023) and with transformers (Qiu et al., 2024), it worked well. Compared to 1-D signal ECG, 2-D ECG images provide more insight into abnormalities and the interpretation of complex cardiac conditions due to their visual representation that combines frequency and temporal characteristics in a single image. Furthermore, when DL architectures are employed, spatial relationships can be exploited to extract important features and improve the classification process:

Recent studies (Yoon and Kang, 2023) have highlighted the importance of scalogram-based approaches for the interpretation of ECG through bimodal CNNs, combined with ensemble and Inception-v3 techniques, achieving an accuracy of 95.08% and 95.74% in classifying ARR, CHF, and NSR, while (Ozaltin and Yeniay, 2023) expanded this work to accurately diagnose COVID-19 with accuracies of 96.53% using CNN and 99.21% with CNN-SVM% respectively.

An extensive investigation has been conducted using ARR, CHF, and NSR datasets to study automatic ECG signal classification, with (Mohamed et al., 2023) reported accuracies of 96%, 92.66%, and 95.33% by using architectures GoogleNet, AlexNet, and ResNet; additionally, Sabeenian and Sree Janani (2023) achieved an accuracy of 98.81% with ResNet18, while combining CNN with Naïve Bayes (Ajjey et al., 2022) and AlexNet (Olanrewaju et al., 2021), reported accuracies of 98.76% and 98.7%, showing the dominance of DL architectures in accurately classifying ECG signals.

Studies using the UCDDB dataset (Mashrur et al., 2021) have applied a scalogram-based CNN to identify obstructive sleep apnea, with an accuracy of 94.30%. Research using the PTB and MIT-BIH arrhythmia datasets (Byeon et al., 2019) has demonstrated the adaptability of DL architectures, including GoogleNet, EECCGNet, and ResNet, attaining high classification accuracies of 92.29% to 99%. Normalizing binary images for extracting the QRS complex (Wang et al., 2020) and with AlexNet, VGG-16, and Inception-V3 (Naz et al., 2021), reflecting ECG properties, such as QRS complexes and T waves, might be advantageous when integrated with other forms of ECG data. Normalization is implemented to achieve consistency in image scaling, which in turn enables the integration of analysis for improved interpretation and detection of cardiac problems.

Recent advancements in ECG analysis have been driven by the exploration of complex DL architectures for enhanced feature extraction and classification. Researchers have leveraged these architectures to improve diagnostic accuracy and enable automated interpretation of ECG signals. Another recent study used 1,932 paper-based ECG images, which were divided into five classes (MI, HMI, NHB, AHB, and COVID-19), to evaluate the performance of different DL architectures in classification tasks. The DenseNet201 (Rahman et al., 2022) model was employed for binary classification, attaining an accuracy of 99.1%. For the classification of three classes, DenseNet201 (Rahman et al., 2022) achieved an accuracy of 97.36%, while InceptionV3 (Rahman et al., 2022) achieved an accuracy of 97.83% for the classification of five classes. A different research study utilized the identical dataset and implemented the In-Res106, InceptionV3, ResNet50, DenseNet201, VGG19, and MobileNetV2 architectures (Fatema et al., 2022) to develop an automated system for predicting heart disease. Impressive levels of accuracy were attained, with In-Res106 emerging as the top performer with a score of 98.34%. In addition, a distinct study that specifically examined the categorization of cardiac disorders (ARR, CHR, NSR) found that the SIFT-CNN (Prusty et al., 2024) attained a remarkable accuracy rate of 99.78%. Nevertheless, alternative techniques such as SVM, K-Nearest Neighbors (KNN), Long Short-Term Memory (LSTM), and AlexNet-SVM (Cnar and Tuncer, 2021) produced diverse outcomes, with accuracy rates ranging from 65.63% to 96.77%, when applied to classification and prediction tasks. A slantlet based feature extraction followed by an SVM classifier has resulted in an Area Under Curve (AUC) of 99.25% (Saeed and Yousif, 2021).

In a different scenario, instead of opting for complex DL architectures, a particular study investigated the use of convolution-based heterogeneous activation facility (CHAF) (Narayanan, 2023). This approach involves employing multiple activation functions (AFs) in the convolution layer blocks, with each block having its own AF. The aim is to extract features more effectively and enhance accuracy. The study achieved an accuracy of 99.55% with an execution time of 0.008 seconds using the CHAF-KNN method on the PTB dataset. Similarly, with the MIT-BIH dataset, the CHAF-KNN method achieved an accuracy of 99.08% and executed in 0.07 seconds.

In similar to our proposed work, there is research work that combines phase and magnitude (Scarpiniti, 2024) of CWT and got 98.5% accuracy; in another work, a time-frequency-based DL framework (Karimulla and Patra, 2025) achieved 94.60% accuracy; and lastly, in another research (Ahmad et al., 2021) uses multimodal image fusion and multimodal feature fusion techniques were used to achieve 99.2%.

Table 1 presents a summary of DL architectures for ECG classification. During our investigation into different DL architectures and ML algorithms for a range of datasets, with the goal of improving feature extraction and model performance in healthcare applications, we have discovered a potential area for innovation: combining the features of two images created from ECG signals.

**Table 1:** Summary of deep learning architectures for ECG classification.

**S. No**.	**Architecture**	**Dataset**	**Accuracy (%)**	**Highlights / Salient Features**
1	1D CNN (Ahmed et al., 2023)	MIT-BIH Arrhythmia Dataset	99.00	Classification into 4 classes
2	1D CNN (Lakhdari and Saeed, 2022)	MIT-BIH Arrhythmia Dataset	97.00–99.00	LeakyReLU activation used
3	SEResNet18 (1D CNN) Nguyen et al. (2022)	ECG image dataset (Cardiac + COVID-19)	98.42 (COVID-19 vs Normal), 98.50 (vs. Other)	Extracted ECG signals from paper records
4	2D CNN (Mewada, 2023)	MIT-BIH Arrhythmia Dataset	99.52	Combines wavelet spectral and temporal CNN features
5	Bimodal CNN (Inception-v3) (Yoon and Kang, 2023)	12-lead ECG (Chapman + Shaoxing)	95.08 (Bimodal), 95.74 (Ensemble)	1D ECG transformed to scalogram and grayscale image
6	CNN–SVM (Ozaltin and Yeniay, 2023)	PhysioNet (ARR, CHF, NSR)	96.53 (CNN), 99.21 (CNN–SVM)	Continuous Wavelet Transform (CWT) used
7	GoogLeNet, AlexNet, ResNet (Mohamed et al., 2023)	PhysioNet (ARR, CHF, NSR)	96.00, 92.66, 95.33	Based on 2D images transformed via CWT
8	ResNet18 (Sabeenian and Sree Janani, 2023)	PhysioNet (ARR, CHF, NSR)	98.81 (raw), 97.05 (wavelet)	1D signal converted to 2D scalogram images
9	CNN + Naïve Bayes (Ajjey et al., 2022)	PhysioNet (ARR, CHF, NSR)	98.76	GoogLeNet used to extract discriminative features
10	AlexNet (Olanrewaju et al., 2021)	PhysioNet (ARR, CHF, NSR)	98.70	1D ECG transformed to 2D scalogram image
11	SCNN (Scalogram-based CNN) (Mashrur et al., 2021)	UCDDB	94.30	Combines Empirical Mode Decomposition (EMD) and CWT
12	GoogLeNet, EECCGNet, ResNet (Byeon et al., 2019)	PTB (CU ECG)	92.29–99.00	CNN-based architecture for ECG images
13	Fusion (AlexNet, VGG19, InceptionV3 + SVM) (Naz et al., 2021)	MIT-BIH Arrhythmia Dataset	97.60	Binary image input with cubic SVM classifier
14	DenseNet201, InceptionV3 (Rahman et al., 2022)	COVID-19 ECG image dataset	99.10 (2-class), 97.36 (3-class), 97.83 (5-class)	Detects COVID-19 from ECG images and signals
15	InRes-106 Hybrid Model (Fatema et al., 2022)	Paper-based ECG images (n=1932)	98.34	Outperforms InceptionV3, ResNet50, etc.
16	SIFT–CNN (Prusty et al., 2024)	PhysioNet (ARR, CHF, NSR)	99.78	Superior to HOG and SURF methods
17	Hybrid AlexNet–SVM (Cnar and Tuncer, 2021)	PhysioNet (ARR, CHF, NSR)	96.77	Utilizes spectrogram representations
18	CNN with heterogeneous activation + KNN (Narayanan, 2023)	PTB, MIT-BIH	99.55 (PTB), 99.08 (MIT-BIH)	Uses six activation functions: tanh, linear, softsign, elu, crelu, relu6
19	CNN-based fusion framework (Scarpiniti, 2024)	MIT-BIH	98.5	Fuses the magnitude and phase of the CWT,
20	Fusion-based time-frequency DL framework (Karimulla and Patra, 2025)	MIT-BIH	94.60	Utilizing spectrograms and scalograms
21	CNN with SVM (Ahmad et al., 2021)	PTB, MIT-BIH	99.2 (PTB), 99.7 (MIT-BIH)	Uses multimodal image fusion and multimodal feature fusion

## 3 Proposed methodology

This section presents a detailed description of the proposed BlendNet architecture, an analysis of BlendNet's computational complexity and an overview of its advantages and limitations.

### 3.1 Proposed BlendNet architecture

**Figure 1:**
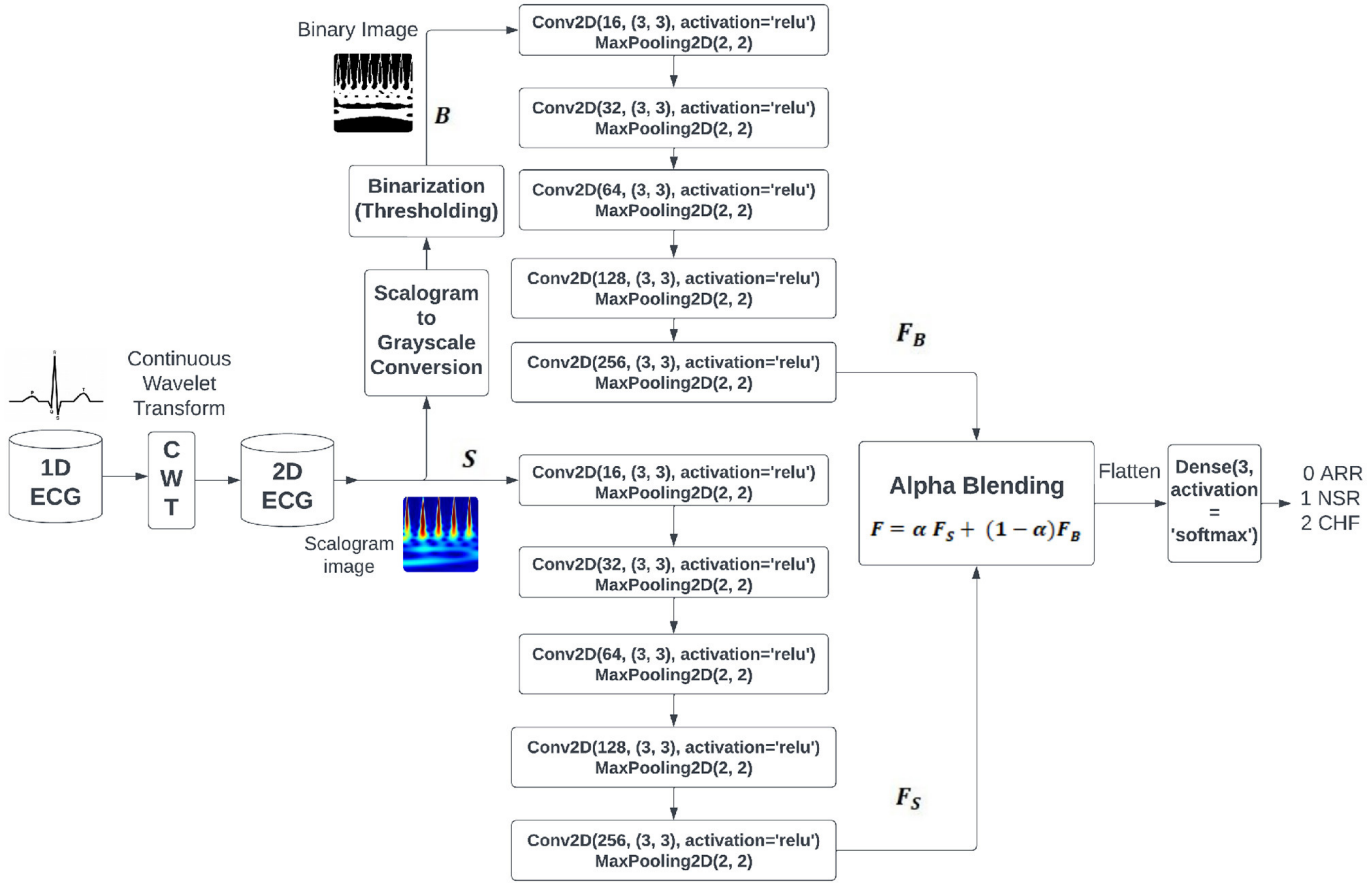
Proposed BlendNet architecture.

Based on the inferences drawn from literature survey, we hypothesize that the composite feature set from different images of the same signal captures fundamental properties of ECG signals more efficiently than the traditional signal representations. In order to achieve this goal, we are incorporating the notion of alpha blending, utilizing its capacity to improve patient care by enhancing diagnostic accuracy and treatment effectiveness. Figure 1 shows the proposed BlendNet architecture. It has three major parts: (i) image formation and binarization, (ii) alpha blending, and (iii) classification.

#### 3.1.1 Image formation and binarization

The continuous wavelet transforms (CWT) play an important role in feature extraction when compared to the 1-D signal, like analyzing nonlinear behavior of ECG signals (Gupta et al., 2021), arrhythmia classification (Kim, 2021), morphological features (Wang et al., 2021), statistical features (Alharbey et al., 2022), performs well with different variants of CNN (Dessai and Virani, 2023), good performance in RGB images (Kumar and Ramachandran, 2023), and classification with transformers (Qiu et al., 2024). It performs a process of signal decomposition, separating the signal into distinct frequency components across a certain time period. Within the context of ECG data, the CWT enables us to examine the signal's time-frequency properties, encompassing both transient and periodic attributes. By utilizing the CWT on the one-dimensional ECG data, we get a two-dimensional representation called a scalogram. The scalogram depicts the temporal changes in the frequency composition of the ECG signal. Every point in the scalogram corresponds to a precise time and frequency, and the intensity of each point represents the magnitude of the wavelet coefficient at that exact time and frequency.

The CWT of the signal f(t) is obtained by integrating f(t) with the shifted and/or scaled forms derived from a mother wavelet Ψ(*t*):


(1) $$\begin{array}{l} CWT\left(a,b\right)=\frac{1}{\sqrt{a}}\int_{-\infty }^{+\infty }f\left(t\right)*{\text{Ψ}}^{*}\left(\frac{t-b}{a}\right)dt \end{array}$$

$$a\in {\mathbb{R}}^{+},\;b\in \mathbb{R}$$

where, *a* is the **scaling parameter** controlling the width of the wavelet transform, *b* is the **translation parameter** controlling the position of the wavelet transform along the time axis, $\text{Ψ}\left(\frac{t-b}{a}\right)$ is the **scaled and translated** version of the **mother wavelet** Ψ(*t*), and Ψ^*^(·) denotes the **complex conjugate** of Ψ(·).


(2) $$\begin{array}{l} CWT\left(\text{scale},\text{position}\right)=\int_{-\infty }^{+\infty }f\left(t\right)\cdot \varphi \left(\text{scale},\text{position},t\right)dt. \end{array}$$

The process of converting the scalogram to grayscale is important in the preprocessing process. This transformation involves various processes like classification of digitized ECG images (Mishra et al., 2021), image-based ECG classification (Li et al., 2021), arrhythmic heartbeat classification (Degirmenci et al., 2022), and bimodal CNN classification (Yoon and Kang, 2023), which entail assigning shades of gray to the intensity levels of the scalogram. This transformation maintains the comparative variations in intensity within the scalogram while streamlining the depiction for subsequent analysis.

Binarisation (i.e., binary conversion) involves simplifying the grayscale representation by applying a threshold to the grayscale image. This binarized image has been employed in various processes, like analysis of QRS complex patterns (Wang et al., 2020), ventricular tachyarrhythmia classification (Naz et al., 2021), and morphological feature extraction for IoT devices (Xiaolin et al., 2022). This procedure entails establishing a threshold value, whereby pixels exceeding this value are designated as white to indicate the existence of a signal, while pixels falling below this value are designated as black to indicate the absence of a signal. The threshold can be chosen based on apriori image information or through techniques such as Otsu's thresholding. The binary image enhances the visibility of the regions of interest in the ECG signal, facilitating the identification of specific features such as peaks, valleys, and anomalies. Table 2 shows the comparison between the two image representations to be considered for the next step in BlendNet.

**Table 2:** Comparison of scalogram and binary image representations for ECG images.

**Aspect**	**Scalogram ECG**	**Binary ECG**
Representation	Time-Frequency domain	Segmentation into foreground/background
Information	Captures time and frequency information	Highlights presence/absence of features
Features	Detailed spectral information	Structural features
Visualization	Spectrogram-like	Black and white
Feature Extraction	Frequency-based	Presence and absence-based

To produce a visualization that combines a scalogram and binary images, the scalogram from the ECG signal will show a depiction of the frequency components of the signal as they change over time. Binary images that depict segmented zones of interest within the ECG signal. This process of segmentation may entail the identification of particular events or irregularities in the signal, such as QRS complexes, P-waves, or T-waves.

#### 3.1.2 Alpha blending

Upon obtaining the scalogram and binary versions of the image, these images are subjected to feature extraction procedure in parallel. As shown in Figure 1, each of these image versions is subjected to a sequence of convolution and pooling layers involving ReLU. The outcomes of these convolution blocks are considered for the alpha blending process. In literature, nighttime single-image dehazing via pixel-wise alpha blending (Yu et al., 2019), content-adaptive feature aggregation mechanism (Fukiage and Oishi, 2021), data hiding in thermal imaging (Rathika and Gayathri, 2021), and no division operation (Van Aken, 2022) are carried out by alpha blending for various purposes. It is a widely employed technique in computer graphics and image processing that combines two images by considering the transparency value (alpha value denoted by α) supplied to each pixel. Alpha blending is a technique used in medical imaging, specifically with ECG, to achieve varied objectives such as overlaying images or annotations, emphasizing specific characteristics, or improving visual representations. It involves assigning an alpha value to each pixel in the input images or layers, indicating its level of transparency or opacity. α values typically span the range of 0 to 1, with 0 representing complete transparency and 1 representing complete opacity. α values ranging from 0 to 1 at an intermediate level produce different degrees of transparency. In BlendNet architecture, the alpha blended feature set is expressed as follows


(3) $$\begin{array}{l} F=\alpha F_{s}+\left(1-\alpha \right)F_{B} \end{array}$$

where, α is the blending proportion, *F*_*S*_ is the feature set obtained from the scalogram version through CNN-type convolution and pooling operations, and *F*_*B*_ is the feature set obtained from the binarized version through convolution and pooling operations, as shown in Figure 1.

#### 3.1.3 Classification using dense layer

The objective of the classification is to differentiate between different classes of input images (i.e., ARR, CHR, and NSR in the cases of PhysioNet datset images). In BlendNet, a dense neural network layer as in conventional CNN is used. Softmax activation function is deployed. This CNN-type dense layer can also be replaced with a more efficient dense layer setting as in InceptionV3, ResNet152V2, DenseNet169, and MobileNetV2. Since computationally more demanding portion of BlendNet is the dense layer, in applications demanding faster convergence, the dense layer can be replaced with ML algorithms such as SVM, Random Forest (RF), KNN, and XGBoost.

### 3.2 Computational complexity of the proposed BlendNet

Let the input image size be *n* × *m* and the convolution kernel size be *k* × *d*. The computational complexity of a convolution operation is O(mnkdf), where *f* denotes the number of filters. If the scalogram and binary images are subjected to *L* layers (convolution + pooling), and the extracted feature map is of dimension *N*, then the total computational complexity of feature extraction followed by alpha blending is O(mnkdfL+N), because the blending requires only O(N) computations. The dense neural network layer (i.e., feedforward neural network) has a computational complexity of $O\left(N^{4}\right)$. Therefore, the computational complexity of the proposed BlendNet is $O\left(mnkdfL+N+N^{4}\right)$. Since *k* ≪ *N*, *d* ≪ *N*, *f* ≪ *N*, and *L* ≪ *N*, the computational complexity can be approximated to $O\left(N^{4}\right)$, which is the same as that of a standard feedforward neural network.

### 3.3 Advantages and limitation of the proposed BlendNet

Salient features of the proposed BlendNet are

- Effective feature extraction: Owing to the fact that each form of the image (i.e., binary and scalogram) contains some unique features of the image, the alpha blending step in the proposed BlendNet facilitates improved feature extraction.
- Flexible blending: The blending is flexible as it is controlled by a parameter α.
- Choice of algorithms for classification: Although the proposed BlendNet architecture contains a dense neural network for classification (as in CNN), it can also be replaced with a more efficient dense-layer or with a faster alternative.

The proposed BlendNet poses a challenge/limitation: The blending proportion α is crucial and it needs to be chosen appropriately.

## 4 Experimental results and discussions

This section contains dataset description, implementation details, definitions of metrics used for evaluation and the experimental results with related discussions.

### 4.1 Dataset description and implementation details

**Figure 2:**
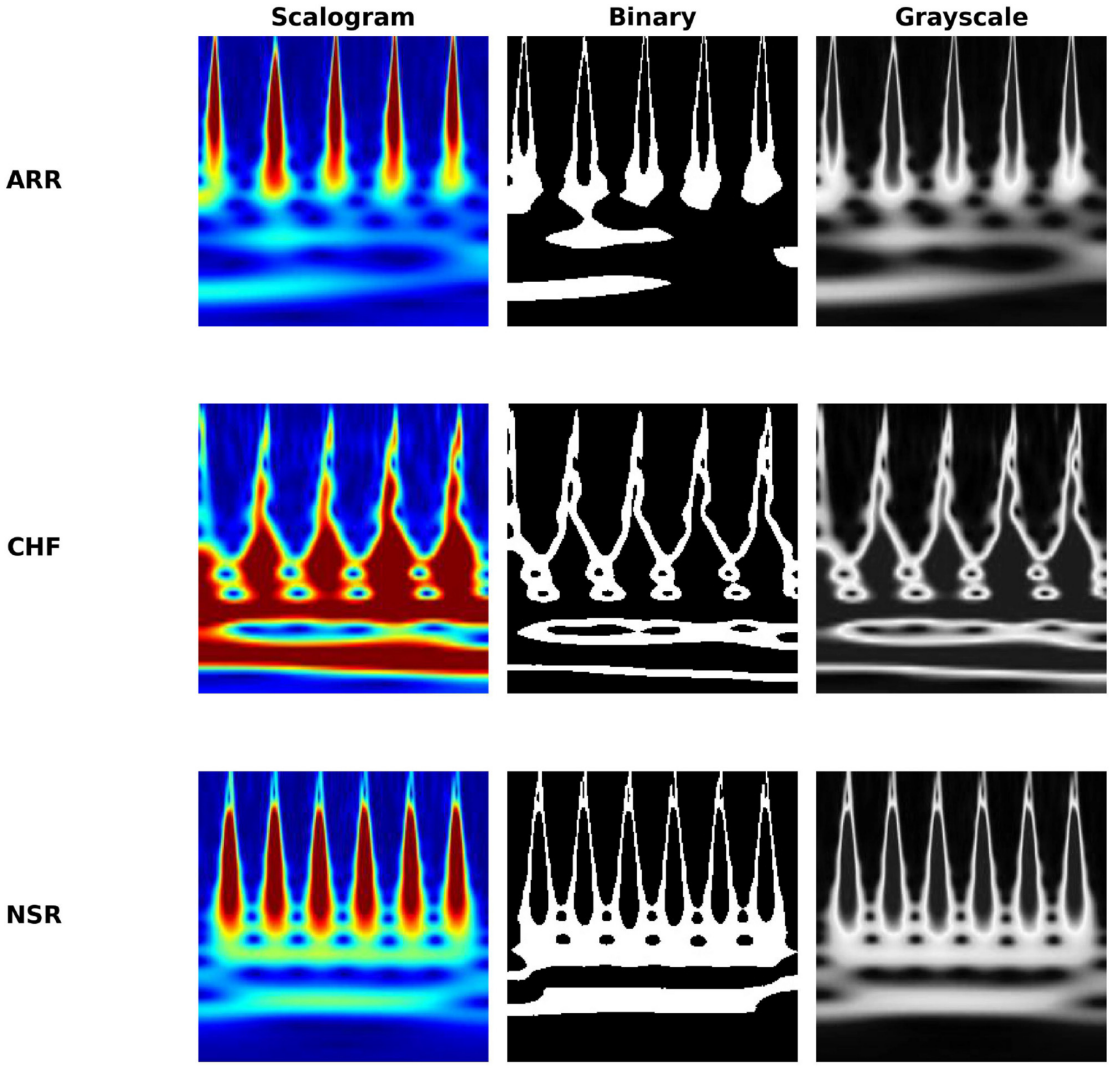
Comparison of ECG representations across cardiac conditions.

Experiments reported in this section utilize ECG data collected from three distinct cohorts: ARR, CHF, and NSR. A total of 162 ECG recordings were utilized, sourced from 3 PhysioNet databases: the MIT-BIH Arrhythmia Database (Moody and Mark, 2001), the MIT-BIH Normal Sinus Rhythm Database (Goldberger et al., 2000), and the BIDMC Congestive Heart Failure Database (Baim et al., 1986). To be more precise, there were 96 recordings from individuals with ARR, 30 recordings from those with CHF, and 36 recordings from individuals with NSR. In Figure 2, which shows the clear visualization of how our ECG looks in all conditions, like ARR, CHF, and NSR, for all image conversions like scalogram, binary, and grayscale images. The objective is to develop a classifier that can accurately differentiate between ARR, CHF, and NSR.

All the ECG signals were processed at a 128 Hz sampling rate. We extracted the first 1,000 samples, and the class distribution is 96 recordings for ARR, 30 for CHF, and 36 for NSR. So 162 ECG signals are used across 3 classes (class-imbalanced dataset) as a pilot study aimed to explore the feasibility of the proposed BlendNet architecture and its ability to extract features. The primary objective was not to achieve state-of-the-art accuracy but to evaluate the effectiveness of our feature extraction pipeline and to analyse the performance trends in a controlled, preliminary setting. The information gained from this work will serve as a foundation for future work involving larger database to validate and generate the findings.

All the data are normalized to zero mean and unit variance. CWT is applied to 1-D ECG into 2-D scalogram images, using the cwtfilterbank MATLAB function. The images have a resolution of 227*227 pixels and are categorized into ARR, CHF, and NSR. The threshold for binarization was fixed 128 because of its simplicity and convention. Technically, pixel values range from 0 to 255; 128 (127.5) is the midpoint. Using a value of 128 effectively separates the morphological features from the background for our dataset and exhibits pixel intensities. We didn't process any data augmentation techniques. During the training step for the model, we applied class_weight to tackle the imbalance condition in the dataset.

As a preliminary experiment, which turned out to be a motivation for proposing BlendNet, we evaluate the performance of CNN models for scalogram and binary images separately, without blending. In our first experiment, we processed scalogram images and binary images separately to the CNN architectures, then by using alpha blending, we blended the images to the dense layer for classification. The BlendNet architecture was implemented for various blending proportions (α ranging from 0.1 to 0.9) and different train-test splits (60:40, 70:30, 80:20, and 90:10). In order to avoid overfitting issues, we have used dropouts in the architectures to mitigate it. Upon identifying the best parameter settings, in the next set of experiments, a slightly modified BlendNet architecture with the CNN-type dense layer replaced with the dense layer settings available in advanced architectures such as InceptionV3, ResNet152V2, DenseNet169, and MobileNetV2 is implemented for a comparative study. Training hyperparameters for the experiments are as follows: we used the “adam” optimiser and “sparse_categorical_crossentropy” loss function, 20 epochs, and a batch size of 64 was processed through the experiments. In the last set of experiments, the BlendNet architecture with the dense layer replaced with faster alternatives (i.e., ML algorithms), such as SVM, RF, KNN, and XGBoost, is implemented.

### 4.2 Evaluation metrics

The experimental study reported in this manuscript uses two key criteria, namely accuracy and execution time, to assess the effectiveness of architectures for ECG classification. Accuracy is a crucial measure for evaluating the efficiency of our architecture in accurately categorizing ECG signals into their appropriate groups. Accuracy is expressed as follows:


(4) $$\begin{array}{l} \text{Accuracy}=\frac{TP+TN}{TP+TN+FP+FN} \end{array}$$

where *TP* is True Positives, *TN* is True Negatives, *FP* is False Positives, and *FN* is False Negatives.

A greater accuracy level signifies good performance in precisely recognizing and diagnosing cardiac problems, hence improving the dependability and practicality of our method. Execution time is a measure of the computing efficiency of our algorithm. It indicates the elapsed time from the start to the end of the architecture's execution (i.e., the computation time in Python 3.9.10 running on a 64-bit AMD Ryzen 7 4800H with Radeon Graphics 2.90 GHz, RAM 16 GB). This measure assesses the amount of computer resources needed to process ECG signals and produce categorization results. Assessing the time spent is vital for evaluating the practical viability and scalability of our approach, especially in real-world situations where prompt diagnosis and decision-making are vital.

### 4.3 Ablation study

In Artificial Intelligence (AI) terminology, an ablation study is done by removing a component/part from an AI model/architecture to understand the importance of the component. Therefore, we start with the ablation study for the proposed BlendNet by analyzing the performance of the CNN architectures on scalogram and binary images separately without blending. The empirical findings, displayed in Table 3, illustrate the classification efficacy of the CNN models for scalogram and binary images. The test train split was fixed as 70:30. The classification based on scalogram images achieved an accuracy of 96.29%, whereas the classification based on binary images achieved an accuracy of 91.11%. The results demonstrate the effectiveness of both image formats in automating ECG classification. The scalogram representation marginally surpasses the binary representation in terms of classification accuracy. The execution time is almost similar.

**Table 3:** Classification performance of traditional approaches for ECG classification.

**Architecture with imaging type**	**Accuracy (%)**	**Execution time (seconds)**
CNN on scalogram images	96.29	272.17
CNN on binary images	91.11	263.24

As there is no blending involved in this experiment, this set of results will serve as a basis for comparison of traditional approaches with the proposed BlendNet architecture which involves blending.

### 4.4 Performance analysis of proposed BlendNet for different blending proportions and for different train-test splits

This experiment focus on examining the efficacy of the proposed BlendNet and the impact of the blending proportion on it. The proportion α is varied from 0.1 to 0.9 (in steps of 0.1). A proportion of 0.1 indicates that 10% of the features are derived from the scalogram and 90% are from the binary counterpart, while a proportion of 0.9 indicates the vice-versa. Table 4 shows the classification performance of BlendNet for different α values and different train-test splits.

**Table 4:** Proposed BlendNet's classification performance for different blending proportions.

**A**	**60:40**		**70:30**		**80:20**		**90:10**	
	**Accuracy (%)**	**Exec. Time (s)**	**Accuracy (%)**	**Exec. Time (s)**	**Accuracy (%)**	**Exec. Time (s)**	**Accuracy (%)**	**Exec. Time (s)**
0.1	96.11	173.51	95.18	203.12	92.22	212.73	96.66	240.01
0.2	94.07	119.99	95.18	130.97	94.44	144.56	93.33	159.47
0.3	92.59	114.36	94.07	131.35	93.88	146.29	95.55	156.48
0.4	95.92	192.70	97.40	195.70	95.00	214.65	94.44	251.64
0.5	93.33	178.49	94.81	190.41	91.66	211.68	96.66	234.81
0.6	94.07	183.30	95.55	206.17	93.33	214.13	96.66	239.09
0.7	100.00	177.43	100.00	197.02	100.00	219.98	100.00	231.67
0.8	95.55	170.02	90.70	192.78	89.44	218.84	96.66	277.94
0.9	93.33	174.75	92.22	214.62	92.77	225.36	93.33	254.37

**Figure 3:**
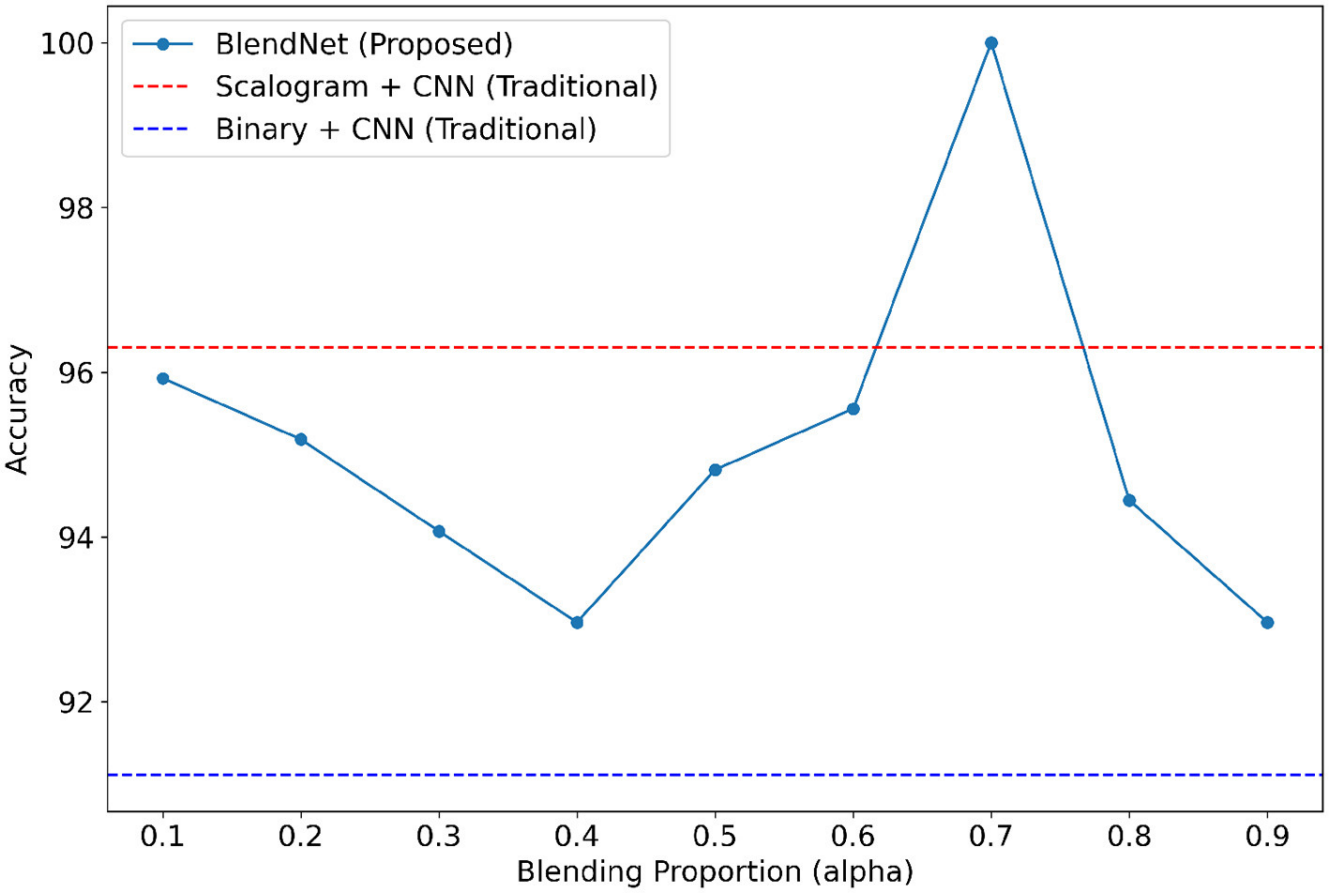
Accuracy of BlendNet vs. blending proportion α.

It can be inferred from Table 4 that the BlendNet's best classification accuracy is achieved for 0.7 regardless of the train-test ratio. Convergence-wise, the architecture is faster for 0.2 or 0.3 compared to other values of α. Figure 3 establishes a comparison of BlendNet's performance with the results obtained from the ablation study. When the proportion deviates from 0.7, the accuracy reduces. The outcome of this experiment indicates that a well-balanced blend of characteristics from both image types resulted in the best categorization performance.

### 4.5 Validation of the robustness of the proposed BlendNet for various dense layer settings

For our study on the robustness of BlendNet in terms of dense layer settings, we replaced the CNN-type dense neural network layer in BlendNet with the dense layer settings available in popular deep learning architectures such as InceptionV3, ResNet152V2, DenseNEt169, and MobileNetV2. Table 5 shows the classification performance of BlendNet with dense layer settings from 4 different architectures, for different α values. The train-test split is fixed as 70:30. It can be inferred from Table 5 that the proposed BlendNet results in a classification accuracy of more than 97% for all 4 dense layer settings considered, with the best accuracy of 99.62% for DenseNet169-type setting. It is worth noting that the highest classification accuracy is achieved for 0.7, regardless of the change in the dense layer setting.

**Table 5:** Proposed BlendNet's classification performance for various dense layer settings.

**α**	**BlendNet-InceptionV3**		**BlendNet-ResNet152V2**		**BlendNet-DenseNet169**		**BlendNet-MobileNetV2**	
	**Accuracy (%)**	**Time (s)**	**Accuracy (%)**	**Time (s)**	**Accuracy (%)**	**Time (s)**	**Accuracy (%)**	**Time (s)**
0.1	90.37	332.90	89.25	1,676.70	94.07	1,287.10	93.70	287.48
0.2	83.33	317.10	90.74	1,704.80	91.85	1,068.00	90.74	286.21
0.3	85.92	637.90	91.11	2,225.60	89.62	1,159.50	91.85	327.62
0.4	85.18	326.60	88.51	1,075.90	91.48	1,298.80	91.48	341.19
0.5	87.03	361.00	91.85	1,705.80	89.99	1,109.40	86.29	294.97
0.6	89.99	315.40	93.70	5,260.90	90.74	1,149.10	89.25	293.13
0.7	97.47	312.60	97.77	1,908.20	99.62	1,112.30	99.25	304.03
0.8	88.88	560.90	92.22	1,785.10	88.51	1,145.40	91.48	332.05
0.9	88.51	374.25	91.85	2,992.90	92.22	1,174.30	92.22	345.90

**Figure 4:**
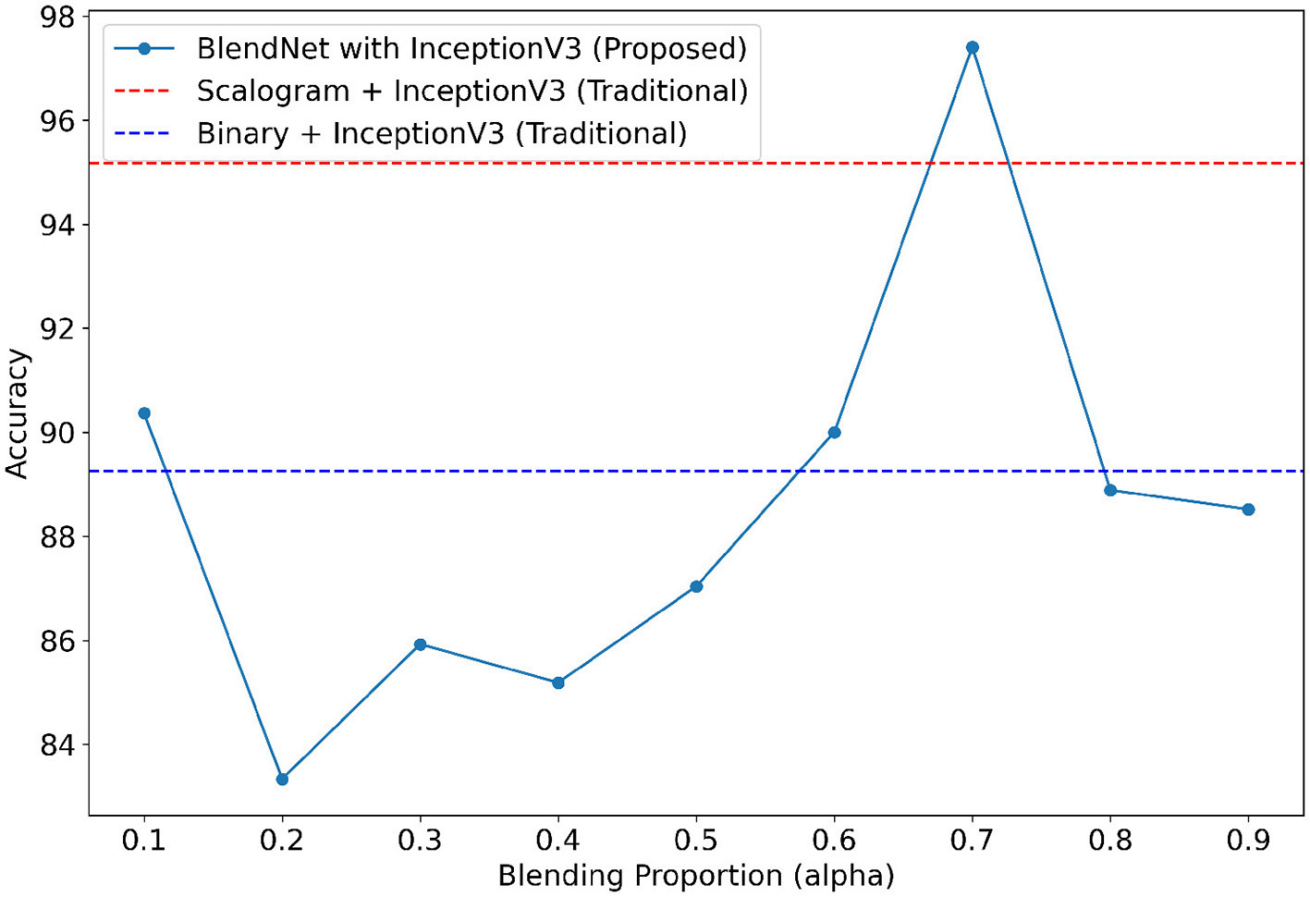
Accuracy of BlendNet-InceptionV3 vs. blending proportion α.

**Figure 5:**
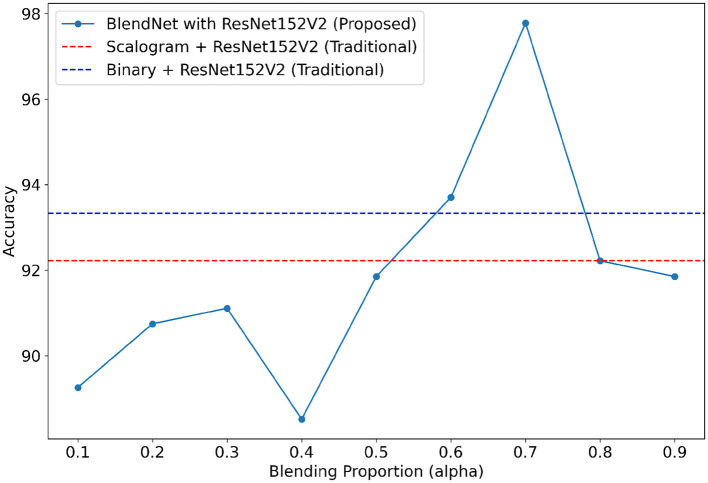
Accuracy of BlendNet-ResNet152V2 vs. blending proportion α.

**Figure 6:**
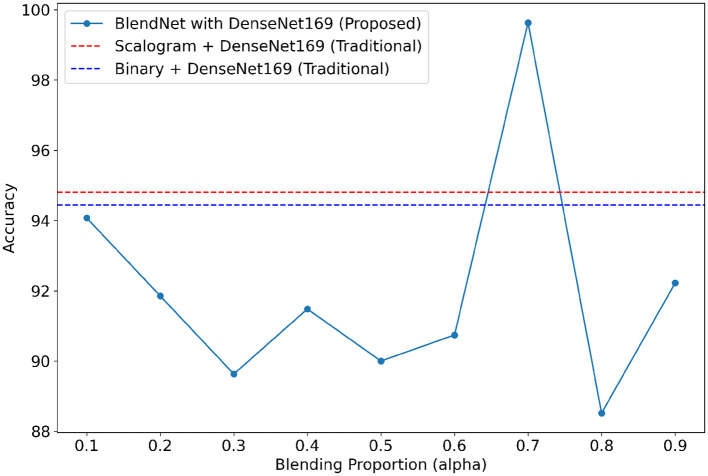
Accuracy of BlendNet-DenseNet169 vs. blending proportion α.

**Figure 7:**
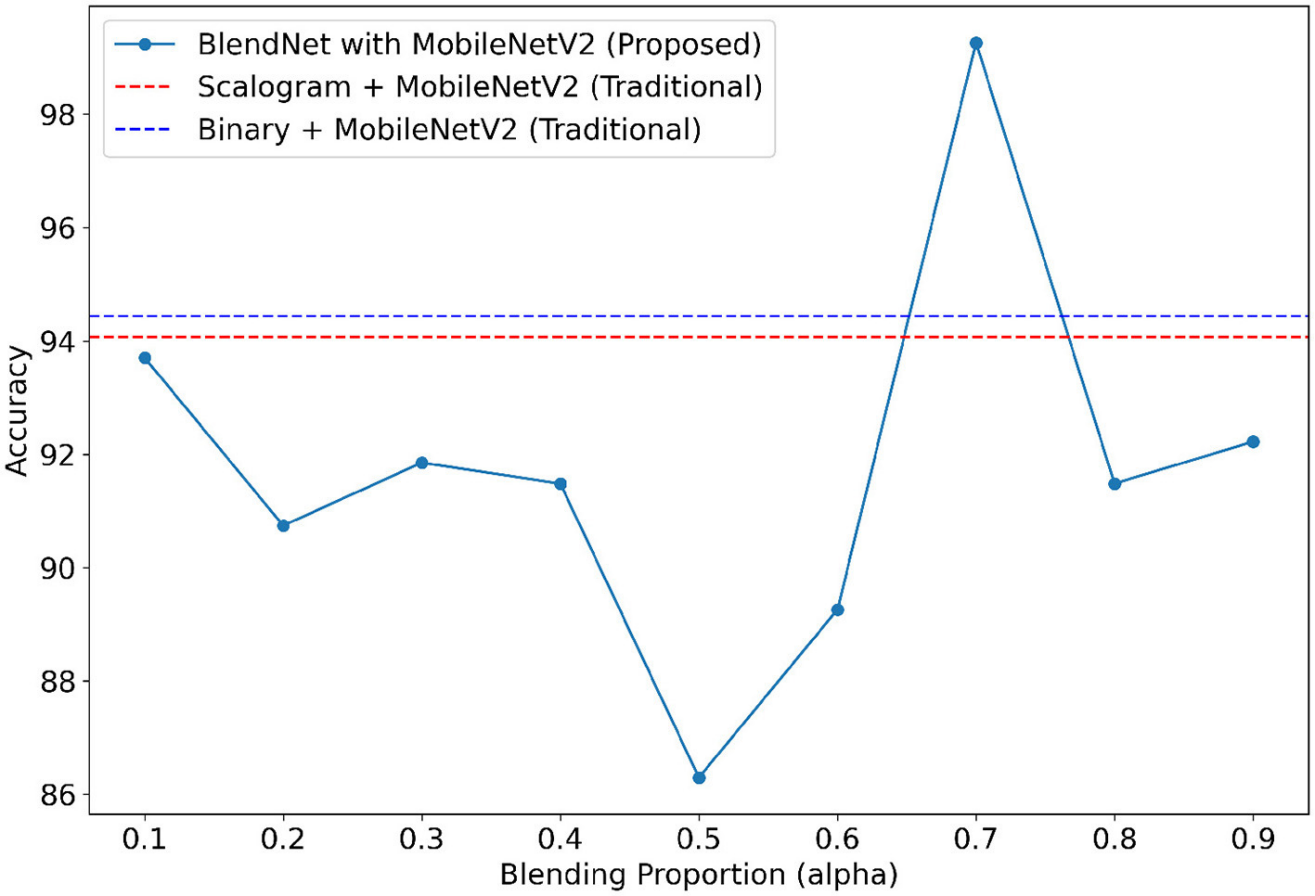
Accuracy of BlendNet-MobileNetV2 vs. blending proportion α.

Convergence-wise, BlendNet with InceptionV3 and MobileNetV2 are comparable to that of the BlendNet with CNN. Figures 4–7 present a comparison of BlendNet's performance against models using only scalogram images and only binary images, without blending. α parameter plays an important role in the blending process; surprisingly, for α = 0.7 proportion, the model (BlendNet, BlendNet-based CNN architecture) gives the best result. When the proportion deviates from 0.7, the accuracy falls below the accuracy. The outcome of this experiment indicates that a well-balanced blend of characteristics from both image types adds flexibility to the proposed BlendNet.

### 4.6 Validation of the robustness of the proposed BlendNet for different ML algorithms

With a motive of providing a faster alternative to the computationally more demanding dense layer in the BlendNet, the dense layer is replaced with ML algorithms. In this experiment, four different ML algorithms—SVM, RF, KNN, and XGBoost are considered. Table 6 shows the classification performance of BlendNet with ML algorithms, for different α values. The train-test split is fixed as 70:30. It can be inferred from Table 6 that the BlendNet's best classification accuracy is achieved for 0.2 regardless of the ML algorithm used. This shows that a binary-dominant blending is more suitable for BlendNet with ML algorithms. Convergence-wise, the architecture is much faster compared to that of the BlendNet architectures involving dense layer.

**Table 6:** Proposed BlendNet's classification performance for different ML algorithms.

**α**	**BlendNet-SVM**		**BlendNet-RF**		**BlendNet-KNN**		**BlendNet-XGBoost**	
	**Accuracy (%)**	**Exec. Time (s)**	**Accuracy (%)**	**Exec. Time (s)**	**Accuracy (%)**	**Exec. Time (s)**	**Accuracy (%)**	**Exec. Time (s)**
**0.1**	97.03	197.31	96.66	200.83	96.29	187.16	96.29	192.24
**0.2**	98.88	133.22	100	130.22	98.88	135.51	99.44	134.71
**0.3**	97.22	134.76	97.22	161.29	97.22	134.64	97.22	136.10
**0.4**	95.18	193.09	95.18	201.13	95.18	220.44	94.81	198.64
**0.5**	96.66	193.34	96.66	202.10	96.66	228.21	97.03	209.98
**0.6**	94.81	193.03	94.81	194.66	94.81	214.45	94.44	204.43
**0.7**	92.22	199.23	92.59	201.26	91.85	198.79	92.22	200.42
**0.8**	93.33	194.79	93.33	204.68	93.33	227.76	93.33	204.98
**0.9**	93.33	194.28	92.96	200.41	93.33	225.55	93.33	205.87

### 4.7 Performance comparison with the state-of-the-art approaches

Table 7 shows the comparison of the proposed BlendNet with the state-of-the-art approaches. The architecture in Prusty et al. (2024) involves SIFT followed by CNN whereas the one in Saeed and Yousif (2021) involves a slantlet transform followed by SVM. It can be inferred from Table 7 that the proposed BlendNet architecture with CNN-type dense layer and α = 0.7 outperforms the state-of-the-art-approaches. To ensure robustness and mitigate overestimation of model performance, we conducted 5-fold stratified cross-validation and got 99.21%±1.05%. The reported metrics are averaged over all folds, with standard deviations included. As mentioned in earlier sections, the main reason behind BlendNet's performance is the composite feature set obtained through alpha blending. In contrast to existing multimodal ECG (Scarpiniti, 2024; Karimulla and Patra, 2025) complex architecture for the fusion process (decision-level fusion with additional classifiers), our proposed BlendNet shows a lightweight and effective linear blending process strategy, enabling transparent and flexible fusion of features from scalogram and binary images. This architecture not only reduces computational cost but also facilitates reproducibility and interpretability.

**Table 7:** Comparison of **BlendNet**'s classification performance with that of the state-of-the-art approaches.

**References**	**Architecture**	**Metrics**
Li et al. (2022)	Slantlet transform + SVM classifier	AUC: 99.25%
Yoon and Kang (2023)	SIFT + CNN (*5-fold* cross validation)	Accuracy: 99.78%
**Proposed**	BlendNet with CNN-type dense layer and α = 0.7	Accuracy: 100%
	BlendNet with RF and α = 0.2	Accuracy: 100%

### 4.8 Effect of alpha blending on feature representation

**Figure 8:**
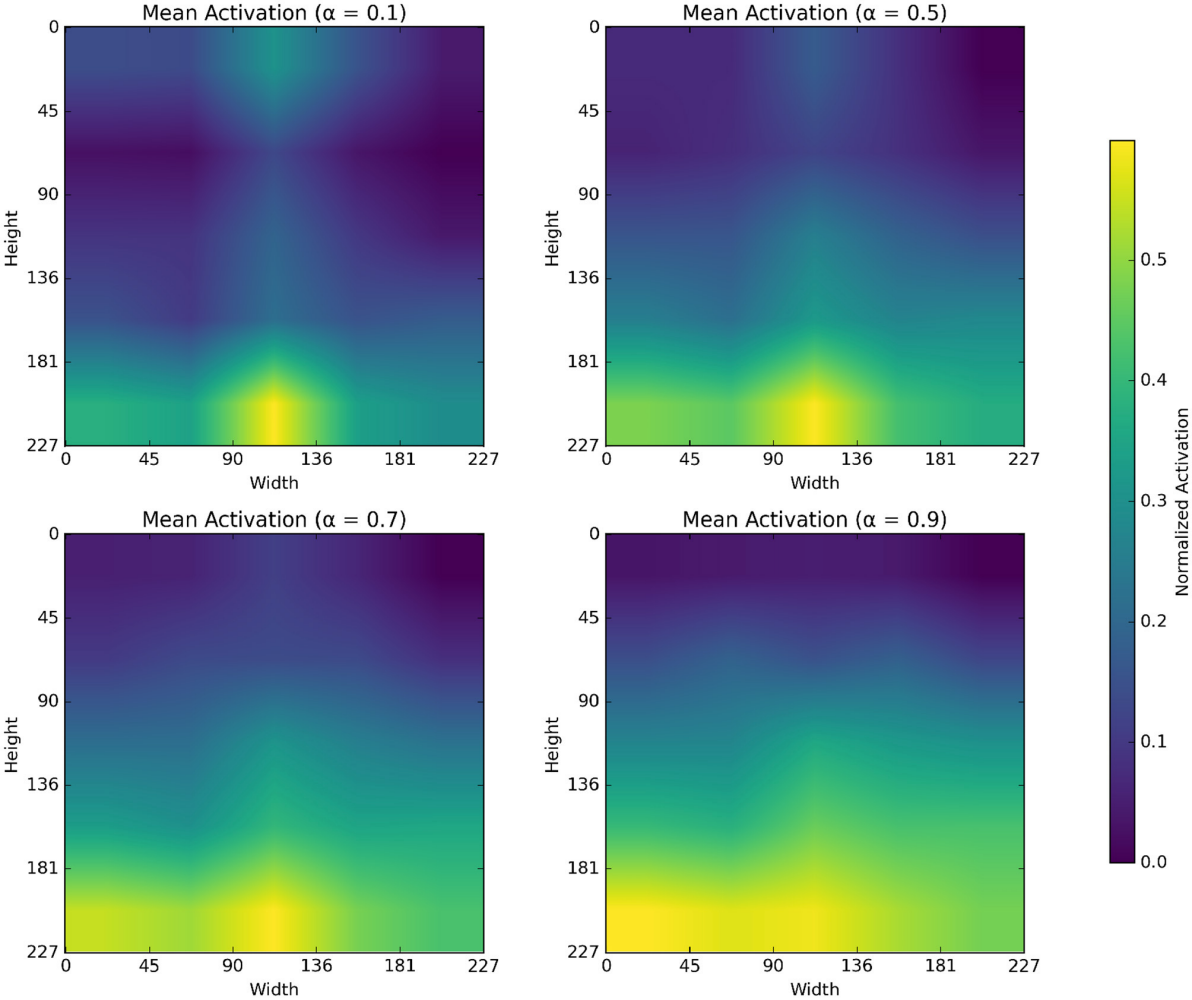
Mean activation heatmap of different α values.

**Figure 9:**
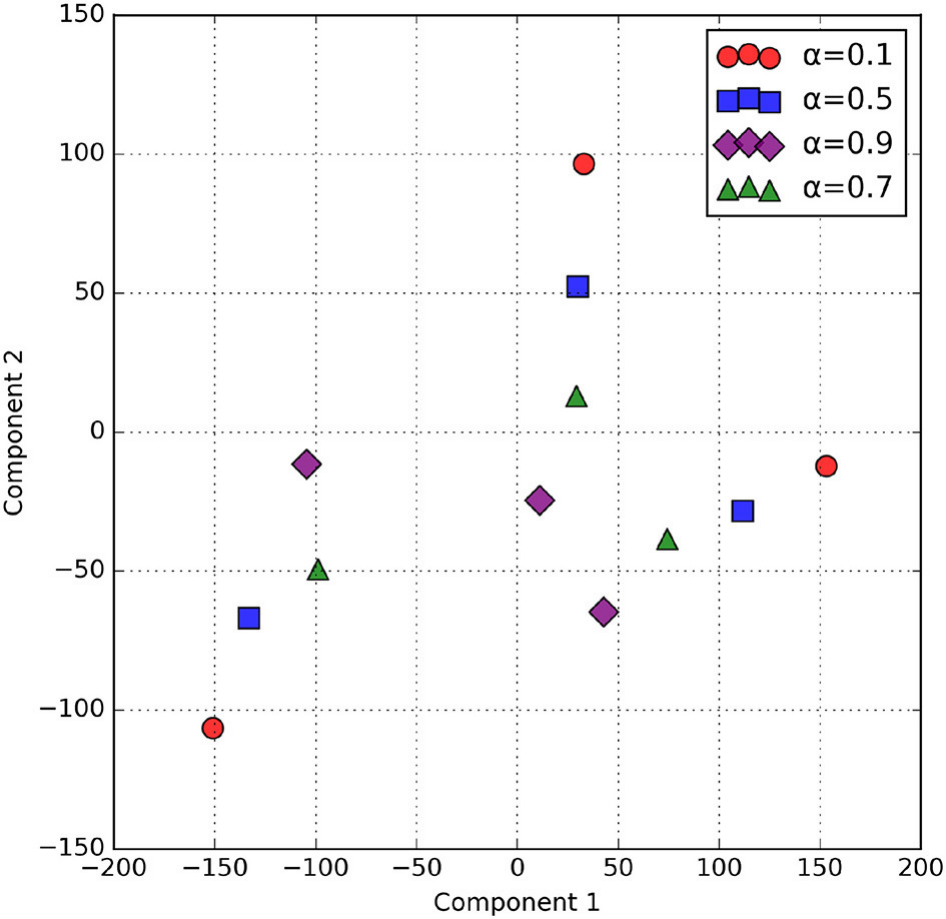
t-SNE of Blended Feature maps across α values.

The α parameter in alpha blending influences both the scalogram and binary image feature representation. Mean activation heatmap, which explains where our model sees the feature for different α values, and t-SNE explains how our α values differences affect feature representation. In Figure 8 for α = 0.1 the blended image, binary CNN features dominate over scalogram features, which means the features are observed less uniformly distributed, like low-level features, whereas for α = 0.9 the blended image, scalogram CNN features dominate over binary features, and the observation is stronger and more spread. For α = 0.5 and α = 0.7, both binary and scalogram features contribute almost equal and moderately strong activation in the central part. From the t-SNE (Figure 9) plot, we calculated the Within-Cluster Sum of Squares (WCSS) for α values like for 0.1, it's 6,134.92; for 0.5, it's 4,338.18; for 0.7, it's 4,117.38, and for 0.9, it's 5,324.92. From this, we can clearly understand that for α = 0.7 tightly clustered meaning, feature representation is good across all the images when compared to all other alpha blending proportions.

## 5 Conclusion

This work proposed “BlendNet,” a novel DL architecture that effectively extracted the features of an ECG signal using a blending approach termed “alpha blending.” The blended feature map is subjected to a dense neural network layer (as in traditional CNN) that classifies the image. The utilization of alpha blending facilitated the generation of a composite feature set that incorporated different characteristics of a 2D-represented ECG signal from its scalogram and binary versions. Experimental results on the PhysioNet dataset showed that the BlendNet has its best performance for α = 0.7. The result of the ablation study showed that, in the case of α = 0.7, BlendNet's performance was better than the performance of its traditional counterparts (i.e., CNN on only scalogram images and CNN only on binarized images). Proposed BlendNet is shown to be flexible in terms of dense layer settings. For applications demanding complicated neural network architectures, BlendNet can be deployed with dense layer settings as in InceptionV3, ResNet152V2, DenseNet169, or MobileNetV2. For applications demanding faster execution times, the dense layer can be replaced with ML algorithms such as XGBoost for faster convergence.

Limitation of the proposed BlendNet: As the blending proportion α deviates from 0.7, the performance starts to degrade. It is also dataset dependent. An approach needs to be devised for estimating an optimal value of α.

Recommendations for future work: (i) As an extension of this work, the effectiveness of blending can be improved by considering other imaging modalities; (ii) The composite feature set resulting from blending can be used in generalized adversarial networks popularly known as GANs.

## Data Availability

Publicly available datasets were analyzed in this study. This data can be found here: https://in.mathworks.com/help/deeplearning/ug/classify-time-series-using-wavelet-analysis-and-deep-learning.html.
